# Investigation of MKRN3 Mutation in Patients with Familial Central Precocious Puberty

**DOI:** 10.4274/jcrpe.5506

**Published:** 2018-07-31

**Authors:** Zehra Aycan, Şenay Savaş-Erdeve, Semra Çetinkaya, Erdal Kurnaz, Melikşah Keskin, Nursel Muratoğlu Şahin, Elvan Bayramoğlu, Gülay Ceylaner

**Affiliations:** 1 University of Health Sciences, Dr. Sami Ulus Obstetrics and Gynecology, Children’s Health and Disease Training and Research Hospital, Clinic of Pediatric Endocrinology, Ankara, Turkey; 2 İntergen Genetic Diagnosis Center, Unit of Genetics, Ankara, Turkey

**Keywords:** MKRN3 mutation, familial central precocious puberty, genetic analysis

## Abstract

**Objective:**

There have been recent advances in the understanding of the etiology of idiopathic central precocious puberty (iCPP) including new genetic associations. The aim of this clinical study was to determine the frequency of *MKRN3* mutation in cases of familial iCPP.

**Methods:**

Potential sequence variations in the maternally imprinted *MKRN3* gene were evaluated in 19 participants from 10 families using next-generation sequencing analysis.

**Results:**

MKRN3 variation was found in only one of the 19 (5.3%) subjects. The male patient, who had a medical history of precocious puberty, had a heterozygous mutation, NM_005664.3:c.630_650delins GCTGGGC (p.P211Lfs*16). The father of this patient also had a history of precocious puberty and had the same mutation. p.P211Lfs*16 is a novel variant and it was identified as probably pathogenic by *in silico* analysis, consistent with the clinical findings.

**Conclusion:**

Given that MKRN3 mutation was detected in only one patient, with a paternal history of precocious puberty, this reinforces the importance of accurate family history taking. The detected incidence of *MKRN3* variants in our case series was much lower than reported elsewhere which suggests a need for further studies in Turkish iCPP patients.

## What is already known on this topic?

Since 2013, the underlying aetiology of some cases of familial idiopathic central precocious puberty (iCPP) has been elucidated.

However, data on the incidence of these new aetiologies of familial iCPP in Turkish populations is scarce.

## What this study adds?

This study showed a low rate of MKRN3 mutation in cases of familial idiopathic central precocious puberty (iCPP) in Turkey.

This case series highlights the importance of always obtaining a good family history when investigating cases of iCPP as this may hasten diagnosis and help identify gene targets for investigation.

## Introduction

Central precocious puberty (CPP) is defined as the development of secondary sex characteristics before eight years of age in girls and nine years of age in boys, due to early activation of the hypothalamic-pituitary-gonadal (HPG) axis (1,2). Owing to recent advances in genetics, the underlying aetiology has been revealed in some cases of idiopathic CPP (iCPP). Gain-of-function mutations in the *KISS1* and *KISSR1* genes and loss-of-function mutations in the makorin ring finger protein 3 (*MKRN3*) gene were shown to result in CPP (3,4,5).

The *MKRN3* gene product exerts an inhibitory effect on gonadotropin releasing hormone (GnRH) neurons. It has been proposed that the HPG axis is reactivated by loss-of-function mutations in the *MKRN3* gene (6). It was reported that the frequency of *MKRN3* was higher in cases with familial iCPP compared with sporadic cases (7,8). However, the frequency can vary according to ethnicity (9). The aim of this clinical study was to determine the frequency of MKRN3 mutation in a group of Turkish families with familial iCPP.

## Methods

The study included siblings diagnosed with iCPP and iCPP cases with a positive family history who presented to the Endocrinology Outpatient Clinic of Dr. Sami Ulus Obstetrics and Gynaecology, Children’s Health and Disease Training and Research Hospital. All parents gave written informed consent before participation. The study was approved by the Ethics Committee of the Zekai Tahir Burak Maternity Teaching Hospital, Ankara, Turkey (46/2015). All children included in the study had at least one first or second degree relative with documented iCPP.

The Tanner and Marshall (10,11) criteria were used for puberty staging. Girls who had at least Tanner stage 2 breast development and stage 2 pubarche before eight years of age were assessed as cases of early puberty. Boys who had at least Tanner stage 2 testicular volume (>4 mL) or stage 2 pubarche before nine years of age were assessed as cases of early puberty.

In the girls luteinising hormone (LH), follicle-stimulating hormone (FSH) and 17β-estradiol (E2) were measured in a morning blood sample. A basal serum LH level ≥0.83 mIU/mL, with puberty precocious findings described above, was accepted as activation of the HPG axis (12). Cases with a basal LH level <0.83 mIU/mL underwent the standard stimulation test of 100 µg GnRH (Ferring Pharmaceuticals, Inc., Parsippany, NJ, USA) by intravenous injection between 8:00 and 8:30 am to assess early puberty. Blood samples were taken at 0, 30, 60, 90, and 120 min to measure serum LH and FSH levels. Peak LH ≥3.3 mIU/mL was accepted as the diagnostic criterion for activation of the HPG axis in girls (13). In boys, LH, FSH and testosterone were measured also in a morning blood sample. A basal serum LH level ≥0.83 mIU/mL, with puberty precocious findings described above, was accepted as activation of the HPG axis in boys (12). Cases with a basal LH level <0.3 mIU/mL underwent the standard stimulation test described above. Peak LH ≥4.1 mIU/mL was accepted as the diagnostic criterion for activation of the HPG axis (13).

Congenital adrenal hyperplasia was excluded by 17-hydroxyprogesterone (17-OHP) <1.5 ng/mL in early morning samples and/or peak 17-OHP <10 ng/mL following an ACTH stimulation test. Cranial magnetic resonance imaging (MRI) was performed to exclude any organic lesion in all cases diagnosed with CPP.

Standing height was measured to the nearest 0.1 cm with a Harpenden fixed stadiometer (Seritex, North America). Body weight was measured on a balance scale (SECA, North America) to the nearest 0.1 kg. Height and weight standard deviation score (SDS) were calculated by comparison with Turkish national reference data (www.ceddcozum.com) (14). Adult height prediction was calculated by dividing the height by the decimal fraction, using the table for predicting adult stature as described by Greulich and Pyle (15).

LH, FSH, and E2 levels were measured with an immunochemiluminometric assay using an Advia Centaur immunoanalyzer (Bayer Diagnostics, Tarrytown, NY, USA). Bone age (BA) was assessed according to the  Greulich and Pyle (15) Atlas method.

### Genetic Analysis

Genomic DNA was isolated from ethylenediamine tetraacetic acid blood sample by Magnesia DNA isolation Kit (Anatolia Geneworks, İstanbul, Turkey). Sequencing study was done by NGS technology and it was performed using the MiSeq next generation sequencing platform (Illumina Inc., San Diego, CA, USA). All coding exons of *MKRN3* and flanking regions were amplified using polymerase chain reaction (PCR) primers, designed with PRIMER-Primer Designer v.2.0 software (Scientific and Educational Software program). Amplicon libraries were prepared with the NexteraXT kit (Illumina Inc.). Sequences were aligned to the hg19 genome with MiSeq Reporter software (Illumina Inc.). Detection of variants was performed with IGV 2.3 (Board Institute) software. *In silico* analysis, database search and literature evaluations were done by Varsome, Polyphen2, HGMD-Public, PubMed, Google search, Clinvar, EXAC and 1,000 Genome studies.

### Statistical Analysis

The data obtained were evaluated using the SPSS 16.0 software programme (SPSS Inc., Chicago, IL, USA).

## Results

The study included 19 patients with CPP from 10 families. In the familial CPP group, there were 17 girls and two boys (one boy with a paternal history of precocious puberty) from 10 families. Clinical, anthropometric and biochemical data of the included patients and their parents are displayed in Tables 1 and 2. Among the 17 girls with familial iCPP, mean age at the onset of secondary sex characteristics was 6.5±1.5 years, and mean age at treatment onset was 7.2±1.4 years. In this group, the mean BA was 8.7±2.0 years, and the BA:CA ratio was 1.2±0.1. The 17-OHP level was normal in all cases with pubarche. Therefore, none of the patients proceeded to an ACTH stimulation test. Cranial MRI was normal in all cases.

**Figure 1:**
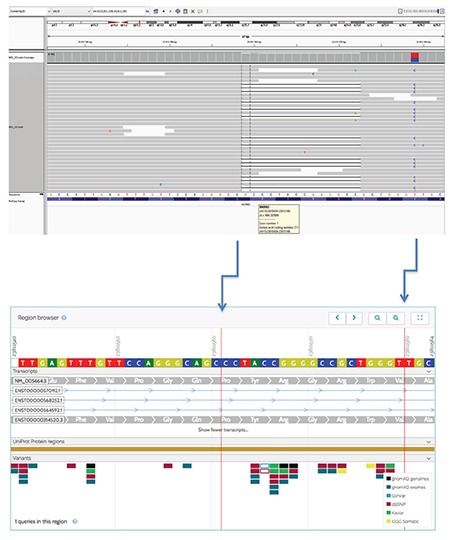
Mutation image of *MKRN3* gene of the patient with IGV2.3 software [NM_005664.3:c.630_650delins GCTGGGC (p.P211Lfs*16)] and Varsome software image

**Figure 2:**
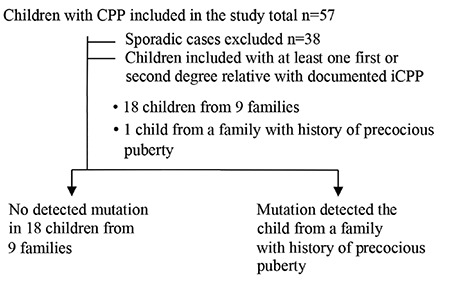
Flow chart of the study recruitment CPP: central precocious puberty, iCPP: idiopathic central precocious puberty

Among the whole group, a novel heterozygous mutation, MRKN3:NM_005664.3:c.630_650delinsGCTGGGC (p.P211Lfs*16), was detected in only one boy with a paternal history of precocious puberty (Figure 1). A flow chart of patient and family recruitment into the study is shown in Figure 2. *MKRN3* gene analysis was performed only in this patient’s father. We did not have the opportunity to study the genotype in his remaining family members. The patient with MKRN3 mutation presented with facial hair growth at 11 years and 7 months of age. Facial hair growth had appeared 1.5 years earlier. Family history revealed that facial hair growth had appeared at the same age in his father. The patient’s brother is unaffected and was found to be pre-pubertal in the examination performed at 10 years of age. The patient’s physical examination yielded the following findings: height, 156.5 cm [+1.27 standard deviation (SD)]; body weight, 44.6 kg (+0.3 SD); 15 mL testicular volume bilaterally; stage 5 pubarche; and axillary hair growth. The heights of mother and father were 167 (+0.6 SDS) and 159 cm (-2.4 SDS), respectively. The patient’s target height and predicted height were estimated to be 169.5 cm (-1 SDS) and 168.8 cm (-1.1 SDS), respectively. Routine biochemistry tests and complete blood count were normal. The hormone test results were as follows: LH, 5.4 mIU/mL; FSH, 13.7 mIU/mL; total testosterone: 393.3 ng/dL; 17OHP: 0.6 ng/mL; and dehydroepiandrosterone sulphate, 60.4 mcg/dL. BA 14 years. The same mutation was also detected in his father. The physical examination of the other boy with no mutation showed height 142.4 cm (1 SDS), weight 35.3 kg (0.6 SDS); 6 mL testicular volume bilaterally; stage 3 pubarche; and axillary hair growth. The heights of mother and father were 149 cm (-2.16 SDS) and 167 cm (-1.3 SDS), respectively. The patient’s target height and predicted height were estimated to be 164.5 cm (-1.65 SDS) and 174 cm (-0.4 SDS), respectively. The patient was followed-up without treatment due to slowly progressive puberty.

This mutation is a frame shift variant and causing production of a truncated protein with 226 amino acids while the wild type protein consists of 507 amino acids. Mutation taster predicts this variant as a disease-causing mutation, probably due to loss of function.

## Discussion

MRKN3, which encodes the *MKRN3*, is an intronless gene located on chromosome 15q11.2 in the Prader–Willi syndrome critical region (16). The imprinted *MKRN3* gene is expressed only in the paternal allele, and it affects both sexes equally, in contrast to female preponderance in iCPP cases (16). The presence of a history of paternal precocious puberty, shorter final height and detection of *MRKN3* gene mutation confirm paternal inheritance. The MRKN3 protein, a product of this gene, includes two copies of a C3H motif in the N-terminal, a novel Cys–His configuration, a C3HC4 RING zinc finger, and a final C3H motif (6). A novel frameshift mutation (between C3H motifs in the N-terminal) in the imprinted *MKRN3* gene was identified in one male case and his affected father. *In silico* analysis suggested that this variant would be pathogenic. Scrutiny of human genetic variant databases revealed that this variant had not been previously reported.

In their study, Abreu et al (5) found a loss-of-function mutation in the *MKRN3 *gene associated with familial iCPP. This work led to an investigation of the mechanism underlying familial iCPP, which has been important not only for understanding iCPP but also for a better understanding of the timing of normal puberty in humans. Since 2013, MKRN3 mutation has been the most frequently identified genetic cause of iCPP. The authors screened 40 individuals with familial iCPP from 15 families for MKRN3 mutations, and reported identifying MKRN3 mutation in 15 individuals from five families (37.5%) (5). In another study, MKRN3 mutation was detected in 13 of 28 cases (46%) with familial iCPP, and in only one of 18 cases with sporadic iCPP (7). In a study of 20 boys with iCPP from 17 families, Bessa et al (17) detected MKRN3 mutation in eight boys from five families. The authors emphasised the importance of investigating boys with MKRN3 mutation and a history of paternal precocious puberty. In a recent study from Turkey, Simsek et al (18) reported that two heterozygous frameshift mutations were identified in the *MKRN3* gene in two probands with familial iCPP and in seven patients with iCPP, as well as 11 unaffected family members. We investigated 19 individuals from 10 families with iCPP and found one novel frameshift (5.3%) mutation. Simsek et al (18) reported that due to the imprinted pattern of inheritance, the phenotype skipped one generation in one family because the proband’s father and paternal uncle had inherited the mutated allele from their mothers. They also showed that in another family, because the proband’s father and affected paternal cousin’s father had inherited the mutated allele from the paternal grandfather, the phenotype was present in the second and third generations. A paternal aunt in the latter family also had iCPP, but her children were asymptomatic carriers of the same mutation. As those authors suggested, and as the history of our patient with MKRN3 mutation highlights, an accurate family history is extremely important, as it can reveal the paternal inheritance of familial iCPP due to a mutation in MRKN3. Physicians should consider this type of inheritance in patients with iCPP thus allowing targeted *MKRN3* genetic analysis, thereby providing an additional tool for the diagnosis of children with iCPP.

In boys, there may be delay in recognising indicators of precocious puberty compared with those (thelarche, menarche) in girls (5,8,9,19,20). The findings of precocious puberty were not recognised by the family in our MKRN3 mutation case, and he presented at the hospital at a late pubertal stage, when he began to shave his facial hair. In the literature, the mean age at onset of puberty was reported as 8.2 years in 13 boys with MKRN3 mutation (5,17,19). Given that age at onset of puberty is approximately six years of age in girls with MKRN3 mutation (5,16,20), pubertal onset appears to be more precocious in affected girls (around two years) than in affected boys (around 0.8 years). In addition, the time from the onset of pubertal symptoms to diagnosis is longer in boys (5,21). It has been reported that puberty can be successfully suppressed by GnRH agonist treatment in cases with MKRN3 mutation and that menarche and other pubertal indicators show a normal course following treatment (5,7,22).

### Study Limitations

The small number of patients and the wide range of criteria which were used to diagnose CPP were the limitations of this study.

## Conclusion

MKRN3 mutation was detected in only one (5.3%) of 19 individuals from 10 families with familial CPP. Given the fact that the MKRN3 mutation was detected in only one patient with a paternal history of precocious puberty in our study, the importance of an accurate family history, which can reveal the paternal inheritance of familial iCPP due to a mutation in MKRN3, must be emphasized. Physicians should consider this type of inheritance in patients with iCPP thus facilitating targeted genetic analysis and providing an additional tool for the diagnosis of children with iCPP.

## Figures and Tables

**Table 1:**
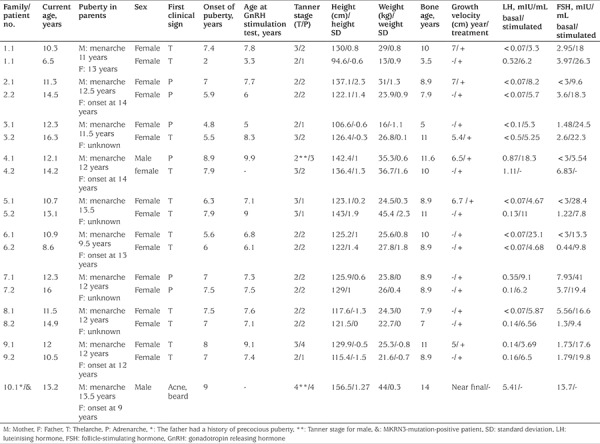
Clinical, anthropometric, and biochemical characteristics of patients

**Table 2:**
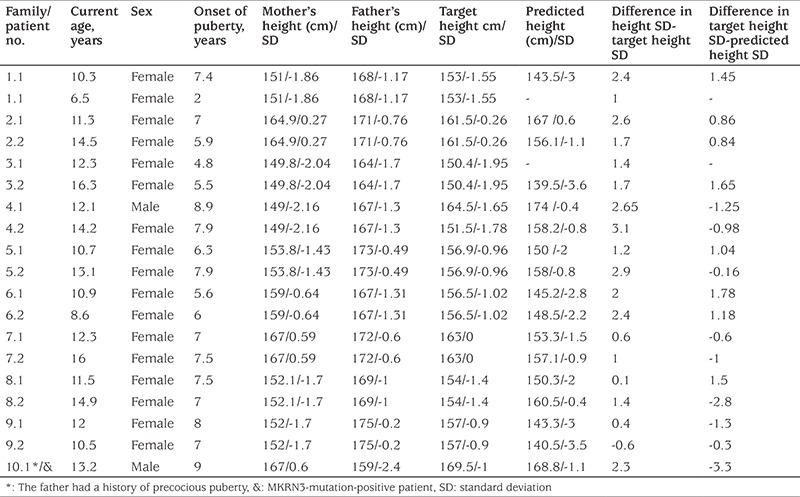
Anthropometric characteristics of patients’ parents, target and predicted height of patients

## References

[1] Diamantopoulos S, Bao Y (2007). Gynecomastia and premature thelarche: a guide for practitioners. Pediatr Rev.

[2] Ibanez L, Virdis R, Potau N, Zampolli M, Ghizzoni L, Albisu MA, Carrascosa A, Bernasconi S, Vicens-Calvet E (1992). Natural history of premature pubarche: an auxological study. J Clin Endocrinol Metab.

[3] Teles MG, Bianco SD, Brito VN, Trarbach EB, Kuohung W, Xu S, Seminara SB, Mendonca BB, Kaiser UB, Latronico AC (2008). A GPR54-activating mutation in a patient with central precocious puberty. N Engl J Med.

[4] Silveira LG, Noel SD, Silveira-Neto AP, Abreu AP, Brito VN, Santos MG, Bianco SD, Kuohung W, Xu S, Gryngarten M, Escobar ME, Arnhold IJ, Mendonca BB, Kaiser UB, Latronico AC (2010). Mutations of the KISS1 gene in disorders of puberty. J Clin Endocrinol Metab.

[5] Abreu AP, Dauber A, Macedo DB, Noel SD, Brito VN, Gill JC, Cukier P, Thompson IR, Navarro VM, Gagliardi PC, Rodrigues T, Kochi C, Longui CA, Beckers D, de Zegher F, Montenegro LR, Mendonca BB, Carroll RS, Hirschhorn JN, Latronico AC, Kaiser UB (2013). Central precocious puberty caused by mutations in the imprinted gene MKRN3. N Engl J Med.

[6] Abreu AP, Macedo DB, Brito VN, Kaiser UB, Latronico AC (2015). A new pathway in the control of the initiation of puberty: the MKRN3 gene. J Mol Endocrinol.

[7] Simon D, Ba I, Mekhail N, Ecosse E, Paulsen A, Zenaty D, Houang M, Jesuran Perelroizen M, de Filippo GP, Salerno M, Simonin G, Reynaud R, Carel JC, Léger J, de Roux N (2016). Mutations in the maternally imprinted gene MKRN3 are common in familial central precocious puberty. Eur J Endocrinol.

[8] de Vries L, Gat-Yablonski G, Dror N, Singer A, Phillip M (2014). A novel MKRN3 missense mutation causing familial precocious puberty. Hum Reprod.

[9] Lee HS, Jin HS, Shim YS, Jeong HR, Kwon E, Choi V, Kim MC, Chung IS, Jeong SY, Hwang JS (2016). Low Frequency of MKRN3 Mutations in Central Precocious Puberty Among Korean Girls. Horm Metab Res.

[10] Marshall WA, Tanner JM (1969). Variations in pattern of pubertal changes in girls. Arch Dis Child.

[11] Marshall WA, Tanner JM (1970). Variations in the pattern of pubertal changes in boys. Arch Dis Child.

[12] Houk CP, Kunselman AR, Lee PA (2009). Adequacy of a single unstimulated luteinizing hormone level to diagnose central precocious puberty in girls. Pediatrics.

[13] Resende EA, Lara BH, Reis JD, Ferreira BP, Pereira GA, Borges MF (2007). Assessment of basal and gonadotropin-releasing hormone-stimulated gonadotropins by immunochemiluminometric and immunofluorometric assays in normal children. J Clin Endocrinol Metab.

[14] Neyzi O, Bundak R, Gökçay G, Günöz H, Furman A, Darendeliler F, Baş F (2015). Reference Values for Weight, Height, Head Circumference, and Body Mass Index in Turkish Children. J Clin Res Pediatr Endocrinol.

[15] Radiographic Atlas of Skeletal Development of the Hand and Wrist, No authors listed (1959). Greulich WW, Pyle SI. California, Stanford University Press,.

[16] Jong MT, Gray TA, Ji Y, Glenn CC, Saitoh S, Driscoll DJ, Nicholls RD (1999). A novel imprinted gene, encoding a RING zinc-finger protein, and overlapping antisense transcript in the Prader-Willi syndrome critical region. Hum Mol Genet.

[17] Bessa DS, Macedo DB, Brito VN, França MM, Montenegro LR, Cunha-Silva M, Silveira LG, Hummel T, Bergadá I, Braslavsky D, Abreu AP, Dauber A, Mendonca BB, Kaiser UB, Latronico AC (2017). High Frequency of MKRN3 Mutations in Male Central Precocious Puberty Previously Classified as Idiopathic. Neuroendocrinology.

[18] Simsek E, Demiral M, Ceylaner S, Kırel B (2017). Two Frameshift Mutations in MKRN3 in Turkish Patients with Familial Central Precocious Puberty. Horm Res Paediatr.

[19] Settas N, Dacou-Voutetakis C, Karantza M, Kanaka-Gantenbein C, Chrousos GP, Voutetakis A (2014). Central precocious puberty in a girl and early puberty in her brother caused by a novel mutation in the MKRN3 gene. J Clin Endocrinol Metab.

[20] Schreiner F, Gohlke B, Hamm M, Korsch E, Woelfle J (2014). MKRN3 mutations in familial central precocious puberty. Horm Res Paediatr.

[21] Hagen CP, Sørensen K, Mieritz MG, Johannsen TH, Almstrup K, Juul A (2015). Circulating MKRN3 levels decline prior to pubertal onset and through puberty: a longitudinal study of healthy girls. J Clin Endocrinol Metab.

[22] Macedo DB, Abreu AP, Reis AC, Montenegro LR, Dauber A, Beneduzzi D, Cukier P, Silveira LF, Teles MG, Carroll RS, Junior GG, Filho GG, Gucev Z, Arnhold IJ, de Castro M, Moreira AC, Martinelli CE Jr, Hirschhorn JN, Mendonca BB, Brito VN, Antonini SR, Kaiser UB, Latronico AC (2014). Central precocious puberty that appears to be sporadic caused by paternally inherited mutations in the imprinted gene makorin ring finger 3. J Clin Endocrinol Metab.

